# Superoxide Dismutase–Centered Modulation by Curcumin in Cardiovascular Diseases: Mechanistic Insights and Translational Implications

**DOI:** 10.1111/jcmm.71233

**Published:** 2026-06-07

**Authors:** Danial Khayatan, Seyed Mehrad Razavi, Zahra Najafi Arab, Amirhossein Niknejad, Yasamin Hosseini, Ayeh Sabbagh Kashani, Saeideh Momtaz, Tannaz Jamialahmadi, Prashant Kesharwani, Amir Hossein Abdolghaffari, Amirhossein Sahebkar

**Affiliations:** ^1^ Department of Toxicology & Pharmacology TeMS.C. Islamic Azad University Tehran Iran; ^2^ GI Pharmacology Interest Group (GPIG), universal Scientific Education and Research Network (USERN) Tehran Iran; ^3^ Medicinal Plants Research Center, Institute of Medicinal Plants, ACECR Karaj Iran; ^4^ Department of Toxicology and Pharmacology School of Pharmacy, and Toxicology and Diseases Group, Pharmaceutical Sciences Research Center (PSRC), the Institute of Pharmaceutical Sciences (TIPS), Tehran University of Medical Sciences Tehran Iran; ^5^ Pharmaceutical Research Center, Pharmaceutical Technology Institute, Mashhad University of Medical Sciences Mashhad Iran; ^6^ Next‐Generation Translational Nanomedicine Laboratory, Department of Pharmaceutical Sciences Dr. Harisingh Gour Vishwavidyalaya (A Central University) Sagar Madhya Pradesh India; ^7^ Applied Biomedical Research Center, Basic Sciences Research Institute, Mashhad University of Medical Sciences Mashhad Iran; ^8^ Centre for Research Impact & Outcome, Chitkara College of Pharmacy, Chitkara University Rajpura India; ^9^ Biotechnology Research Center, Pharmaceutical Technology Institute, Mashhad University of Medical Sciences Mashhad Iran

**Keywords:** cardiovascular disease, curcumin, nanodelivery, oxidative stress, superoxide dismutase

## Abstract

Cardiovascular diseases (CVD) remain the leading global cause of morbidity and mortality, driven in part by dysregulated redox homeostasis and chronic inflammation. Superoxide dismutase (SOD), a key enzymatic defence against reactive oxygen species (ROS), plays a central role in maintaining cardiovascular integrity through regulation of oxidative stress across cytosolic (SOD1), mitochondrial (SOD2) and extracellular (SOD3) compartments. Impairment of SOD function contributes directly to endothelial dysfunction, myocardial injury and vascular remodelling. Curcumin (Cur), a pleiotropic polyphenol derived from *Curcuma longa* , has emerged as a potent modulator of SOD activity and expression. Evidence from preclinical models consistently demonstrates that Cur enhances SOD‐dependent antioxidant defences, thereby attenuating oxidative damage, inflammation, apoptosis and fibrosis across multiple CVD contexts, including myocardial infarction, cardiomyopathy, hypertension and diabetic complications. While Cur also influences additional signalling pathways, such as NF‐κB, PI3K/AKT and Nrf2, these effects are increasingly understood to converge on SOD‐mediated redox regulation. Recent advances in nanodelivery systems have further improved Cur bioavailability and its capacity to modulate SOD activity in vivo. However, despite robust preclinical evidence, clinical validation remains limited. This review synthesizes current mechanistic and translational evidence, positioning SOD as the central mediator of Cur's cardioprotective effects and highlights key gaps in clinical translation.

## 1 Introduction

Cardiovascular diseases (CVD) constitute the most formidable global health burden and are projected to exceed 23 million deaths annually by 2030 [1, 2]. Their rising prevalence reflects a complex interplay of metabolic, inflammatory, genetic and environmental drivers [3]. While traditional risk factors, including dyslipidemia, hypertension, diabetes, sedentary lifestyle and population aging, remain central, a unifying molecular hallmark across CVD is oxidative stress, arising from excessive production of reactive oxygen species (ROS) and impaired antioxidant defences [4, 5]. ROS are indispensable for physiological signalling, yet pathological ROS accumulation disrupts mitochondrial respiration, damages membrane lipids, oxidizes proteins and mutates DNA [4, 5]. The cardiovascular system is uniquely sensitive to redox imbalance, with perturbations contributing to endothelial dysfunction, cardiomyocyte death, maladaptive remodelling and inflammatory amplification [6, 7, 8]. Among endogenous antioxidant defences, superoxide dismutase (SOD) occupies a pivotal position as the first‐line enzymatic barrier against superoxide radicals. Its three mammalian isoforms, SOD1 (cytosolic), SOD2 (mitochondrial) and SOD3 (extracellular), orchestrate ROS detoxification across cellular compartments [9, 10, 11, 12]. Deficiencies or dysfunction of SOD isoforms have been mechanistically linked to atherosclerosis progression, ischemia–reperfusion injury, hypertensive vascular remodelling, diabetic microvascular disease and adverse outcomes in cardiomyopathy. Thus, restoring SOD activity represents a compelling therapeutic strategy for modifying the redox landscape underlying CVD [13]. Curcumin (Cur), the principal bioactive component of *Curcuma longa* , displays pleiotropic antioxidant, anti‐inflammatory and immunomodulatory properties [14, 15, 16, 17, 18]. Importantly, Cur upregulates SOD expression, enhances enzymatic activity, stabilizes mitochondrial redox balance and modulates nuclear factor‐erythroid factor 2‐related factor 2 (Nrf2), nuclear factor‐kappa B (NF‐κB), phosphatidylinositol‐3 kinase (PI3K)/protein kinase B (AKT)/rapamycin target protein (mTOR) and mitogen‐activated protein kinase (MAPK) pathways, mechanisms intimately involved in cardiovascular injury and repair [19, 20]. Although Cur's clinical translation has historically been hampered by limited aqueous solubility and rapid metabolism, modern nanodelivery platforms now achieve markedly improved bioavailability and targeted delivery [21, 22, 23], with the potential of enhanced SOD modulation and cardiovascular protection. In this context, SOD represents a central mechanistic node linking oxidative stress, inflammation and cellular injury in CVD. Therefore, rather than broadly examining curcumin as a general antioxidant, this review specifically focuses on SOD‐centered mechanisms, emphasizing how curcumin modulates SOD isoforms, enzymatic activity and downstream redox signalling pathways across different cardiovascular conditions.

## 2 Method of Research

Data for this narrative review were collected from Scopus, Google Scholar, PubMed and the Cochrane Library, focusing on clinical, in vivo and in vitro studies published in English from 1969 through December 2025. A comprehensive search strategy was employed using combinations of the following terms: ‘cardiovascular diseases’, ‘cardiovascular disorders’, ‘superoxide dismutase’, ‘SOD’, ‘myocardial infarction’, ‘cardiomyopathy’, ‘hypertension’, ‘cardiac toxicity’, ‘abdominal aortic aneurysm’, ‘diabetes mellitus’, ‘curcumin’ and ‘ *Curcuma longa* .’ Boolean operators (AND/OR) were applied to identify studies evaluating the relationship between curcumin, SOD activity, oxidative stress and cardiovascular pathology. Studies were included if they (i) investigated curcumin or its derivatives, (ii) assessed oxidative stress or antioxidant pathways with specific reference to SOD activity or expression and (iii) reported outcomes relevant to cardiovascular or cardiometabolic conditions. Additional relevant articles were identified through manual screening of reference lists. Only peer‐reviewed publications containing mechanistic, pharmacological or therapeutic insights were included. Reviews, editorials and non‐English publications were excluded unless they provided essential background information. Overall, the review includes approximately 12 in vitro studies, 15 in vivo studies and a limited number of clinical investigations, reflecting the current distribution of evidence in this field. The majority of included studies were preclinical, with relatively few clinical trials directly assessing cardiovascular outcomes. No formal quality assessment tools (e.g., risk‐of‐bias scoring systems) were applied, as this work was conducted as a narrative review rather than a systematic review. This represents a limitation and may introduce selection and interpretation bias.

## 3 Pharmacological Applications of Curcumin

**FIGURE 1:**
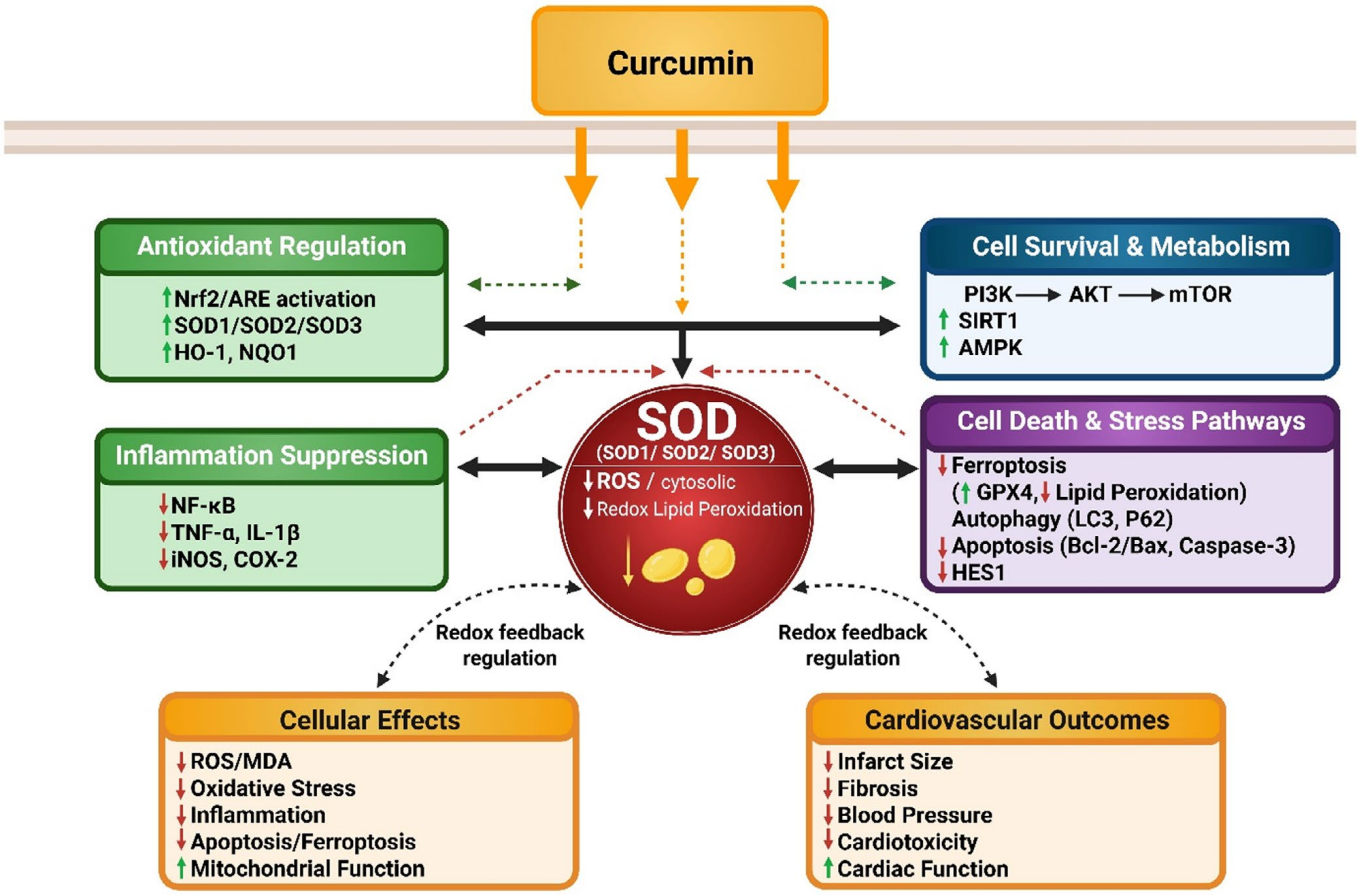
Cur‐mediated modulation of SOD‐centered redox and signalling networks in CVD. Curcumin (Cur) exerts cardioprotective effects through coordinated regulation of multiple interconnected signalling pathways that converge on superoxide dismutase (SOD) isoforms (SOD1, SOD2 and SOD3), which serve as a central hub of redox homeostasis. Cur activates antioxidant pathways via nuclear factor erythroid 2–related factor 2 (Nrf2)/antioxidant response element (ARE) signalling, leading to increased expression of SOD and downstream cytoprotective enzymes, including heme oxygenase‐1 (HO‐1) and NAD(P)H quinone dehydrogenase 1 (NQO1). Concurrently, Cur suppresses inflammatory signalling by inhibiting nuclear factor‐kappa B (NF‐κB) and reducing pro‐inflammatory mediators, such as tumour necrosis factor‐α (TNF‐α), interleukin (IL)‐1β and inducible nitric oxide synthase (iNOS). Cur further modulates cell survival and metabolic pathways through activation of phosphatidylinositol‐3 kinase (PI3K)/protein kinase B (AKT)/mammalian target of rapamycin (mTOR), Sirtuin 1 (SIRT1) and AMP‐activated protein kinase (AMPK). In parallel, Cur attenuates stress‐induced cell death mechanisms, including ferroptosis, autophagy and apoptosis, partly through regulation of glutathione peroxidase 4 (GPX4), BCL2‐associated X protein (Bax) and hairy and enhancer of split 1 (HES1). These pathways collectively regulate SOD activity, leading to reduced superoxide (O_2_^−^) and reactive oxygen species (ROS) accumulation, decreased lipid peroxidation and restoration of redox balance. SOD‐mediated redox regulation subsequently exerts feedback control on upstream signalling pathways, reinforcing antioxidant and anti‐inflammatory responses. These molecular effects translate into improved cellular outcomes, including reduced oxidative stress, inflammation, apoptosis and mitochondrial dysfunction, ultimately leading to favourable cardiovascular outcomes such as decreased infarct size, fibrosis, blood pressure and cardiotoxicity, along with improved cardiac function.

Cur is a bioactive polyphenolic compound derived from *Curcuma longa* of the Zingiberaceae family. It is highly soluble in organic solvents but poorly soluble in aqueous environments, although its solubility increases under alkaline conditions. Cur has demonstrated therapeutic potential across a broad spectrum of diseases, including cancer, CVD, inflammatory bowel disease, neurodegenerative impairments and diabetes [24, 25, 26, 27, 28]. Its anti‐inflammatory activity is largely attributed to the suppression of key pro‐inflammatory mediators—such as interleukin (IL)‐1β, IL‐12, tumour necrosis factor‐α (TNF‐α), chemokines and NF‐κB [29, 30, 31, 32]. Additionally, the antioxidant effects of curcumin arise both from upregulation of endogenous defence systems (e.g., SOD, glutathione enzymes) and from the direct reduction of ROS and RNS generation [33, 34, 35, 36]. Despite its favourable safety profile in humans, Cur's therapeutic implementation is hindered by inherently low bioavailability due to low gastrointestinal absorption, rapid metabolism and limited systemic distribution [37]. To overcome these limitations, a variety of advanced delivery systems, including nano‐ and micro‐formulations, liposomal encapsulation, micelles and co‐administration with bioavailability enhancers, have been developed [38, 39, 40]. Nanoparticle (NPs)‐based delivery has emerged as a particularly effective strategy, offering protection against metabolic degradation, improved tissue targeting and controlled drug release. Liposomal systems similarly enhance aqueous solubility and stability, while implantable or sustained‐release platforms provide additional improvements in pharmacokinetic performance [23]. A mechanistic figure of CUR is illustrated to better elucidate its therapeutic effects via several signalling pathways in different disorders (Figure 1).

## 4 Intervention of Cur via SOD in Different CVD

### 4.1 Myocardial Infarction

Myocardial infarction (MI) results from acute obstruction of coronary blood flow, typically due to plaque rupture and thrombus formation involving lipids, inflammatory cells and extracellular matrix components. Impaired perfusion initiates ischemic injury and cardiomyocyte necrosis, manifesting clinically as chest pain, dyspnea, diaphoresis, nausea, vomiting, arrhythmia and fatigue. Notably, up to 64% of cases may present without chest pain, termed silent MI [41, 42]. Established risk factors, including advanced age, male sex, family history, tobacco use, elevated low‐density lipoprotein (LDL), obesity, physical inactivity, alcohol excess and illicit drug use, further accelerate disease progression [43]. A central feature of MI pathophysiology is excessive reactive oxygen species (ROS) generation together with impairment of endogenous antioxidant defences, particularly superoxide dismutase (SOD). Reduced SOD activity during ischemia and reperfusion amplifies lipid peroxidation, mitochondrial dysfunction and cardiomyocyte loss, thereby worsening myocardial injury [44]. Consistent with this, higher SOD activity has been associated with improved myocardial recovery [45]. Cur exerts cardioprotective effects in MI predominantly through restoration of SOD‐dependent redox homeostasis. In vitro, Cur (5–20 μM) attenuated hypoxia/reoxygenation injury by increasing SOD expression, reducing malondialdehyde (MDA) levels, suppressing ROS accumulation and improving cell viability, with these effects occurring in parallel with inhibition of the mitogen‐activated protein kinase (MAPK) pathway [46]. In vivo, Cur (50 mg/kg) reduced cardiac biomarkers, including lactate dehydrogenase (LDH), creatine kinase (CK) and cardiac troponin I (cTnI), while also decreasing MDA, apoptosis, necrosis and collagen deposition, collectively improving post‐MI remodelling [47]. Additional evidence showed that Cur (1.5–200 μg/mL) increased SOD and glutathione (GSH) activity, reduced MDA and nitric oxide (NO), decreased BCL2‐associated X protein (Bax) and infarct size and increased B‐cell lymphoma 2 (Bcl‐2) expression. These effects were associated with activation of the phosphatidylinositol‐3 kinase (PI3K)/protein kinase B (AKT)/mammalian target of rapamycin (mTOR) pathway [48]. More recent mechanistic work further strengthens the SOD‐centered role of Cur in ischemia/reperfusion injury. Yuan et al. showed that Cur pretreatment (10 μM) markedly attenuated anoxia/reoxygenation‐induced damage in H9c2 cardiomyocytes by suppressing ferroptosis, oxidative stress, excessive autophagy and apoptosis through upregulation of hairy and enhancer of split 1 (HES1). These protective effects were accompanied by restoration of SOD activity, improvement in the glutathione/glutathione disulfide (GSH/GSSG) ratio, reduction of MDA and intracellular iron accumulation and increased glutathione peroxidase 4 (GPX4), thereby linking Cur‐mediated SOD restoration to broader redox‐protective signalling [44].

Complementing these mechanistic findings, an in vivo MI model showed that Cur‐loaded PEGylated graphene quantum dots (Cur‐PEG‐GQDs) significantly improved hemodynamic parameters, reduced infarct size and attenuated myocardial fibrosis compared with free Cur. Cur‐PEG‐GQDs restored systolic blood pressure (SBP), diastolic blood pressure (DBP), left ventricular systolic pressure (LVSP), the maximum rate of left ventricular pressure rise (+dP/dt) and the maximum rate of left ventricular pressure decline (−dP/dt), while lowering MDA and partially restoring the reduced‐to‐oxidized glutathione ratio. Although Cur‐PEG‐GQDs showed complex dose‐dependent effects on SOD, total antioxidant capacity (TAC) and glutathione peroxidase (GPX), these findings suggest that nanocarrier delivery may enhance the cardioprotective efficacy of Cur in MI [49].

Although these findings strongly support the cardioprotective effects of Cur in MI, most evidence is derived from in vitro models or small‐scale animal studies. SOD activity is consistently increased in parallel with ROS; however, it is typically measured as a global antioxidant endpoint rather than through isoform‐specific or mechanistic targeting. Therefore, while SOD restoration appears to play a key role in mediating curcumin's effects, its precise causal contribution to myocardial protection remains to be fully established.

### 4.2 Cardiomyopathy

Cardiomyopathies encompass hypertrophic, dilated and restrictive subtypes and may be classified as primary (genetic or acquired) or secondary to systemic disease processes [50]. Clinical manifestations, including edema, fatigue, orthopnea, dyspnea, syncope and ischemia, reflect impaired myocardial structure and function. Oxidative stress is a major pathogenic driver in cardiomyopathy, as excessive ROS generation and mitochondrial dysfunction promote inflammation, apoptosis, fibrosis and adverse remodelling. Diminished antioxidant defences, particularly reduced SOD activity, further exacerbate myocardial injury, whereas increased SOD and Sirtuin‐1 expression have been associated with improved outcomes [51, 52]. Various natural products have been identified as potential therapeutic agents against different types of cardiomyopathy [53, 54, 55]. Cur exerts protective effects in cardiomyopathy largely through modulation of SOD‐centered antioxidant pathways. Ren et al. demonstrated that Cur (100 mg/kg) attenuated myocardial dysfunction by activating PI3K/AKT and Sirtuin‐1 signalling while reducing Forkhead box protein O1 (Foxo1) acetylation, leading to increased SOD activity, decreased MDA levels, reduced Bax and caspase‐3 and increased Bcl‐2, nuclear factor erythroid 2–related factor 2 (Nrf2) and NAD(P)H quinone dehydrogenase 1 (NQO1) expression [56]. Similarly, tetrahydroCur (120 mg/kg) reduced oxidative stress, myocardial fibrosis and hypertrophy while increasing SOD2, GSH and Sirtuin‐1 and decreasing transforming growth factor‐beta 1 (TGFβ1)/Smad3, α‐smooth muscle actin (α‐SMA) and collagen I/III expression [57]. Another study showed that Cur (200 mg/kg) elevated SOD, GSH and catalase (CAT) while reducing MDA and lowering cardiac injury markers, including alkaline phosphatase (ALP), aspartate aminotransferase (AST) and alanine transaminase (ALT) [58]. Some models also revealed dose‐dependent responses, with Cur (20 mg/kg) activating SOD and Nrf2/antioxidant response element (ARE) signalling despite mild increases in oxidative stress markers [59]. Collectively, these studies indicate that Cur improves cardiomyopathy phenotypes primarily through strengthening SOD‐linked antioxidant defences, while changes in fibrosis, apoptosis and inflammatory signalling appear to occur downstream of redox restoration [60, 61, 62]. Additional evidence implicates SOD modulation in Cur's cardioprotective effects against toxic cardiomyopathy. Hamdy et al. showed that Cur (200 mg/kg) significantly ameliorated gentamicin‐induced cardiac toxicity by restoring SOD, CAT and GSH activities and reducing MDA accumulation in myocardial tissue. Cur also downregulated pro‐inflammatory mediators, including NF‐κB, interleukin (IL)‐1β, Kelch‐like ECH‐associated protein 1 (Keap1), heme oxygenase 1 (HMOX1) and Bax, while upregulating Nrf2 and Bcl‐2 expression. These molecular improvements corresponded with attenuation of cardiac fibrosis, leukocyte infiltration and myofiber degeneration, further supporting Cur's ability to limit cardiomyopathic injury through the SOD/Nrf2 redox axis [63]. Collectively, these studies demonstrate that Cur improves cardiomyopathy outcomes through modulation of oxidative stress and SOD‐related pathways. However, most investigations rely on rodent models and biochemical endpoints without directly interrogating SOD isoform‐specific mechanisms. Thus, SOD is best interpreted as a central component of the antioxidant response rather than an independently validated mechanistic driver in cardiomyopathy.

### 4.3 Hypertension

Hypertension is a chronic elevation of arterial blood pressure resulting from increased cardiac output and/or vascular resistance. Although often asymptomatic, it may present with headache, dyspnea or epistaxis [64]. Major risk factors include aging, genetic predisposition, obesity, inactivity, family history, tobacco use, high sodium intake, low potassium intake and excessive alcohol consumption. Inflammation and oxidative stress are important contributors to hypertension and excessive ROS generation disrupts endothelial function while suppressing endogenous antioxidant enzymes, including SOD, thereby amplifying vascular injury [65, 66]. Cur and its metabolites have demonstrated antihypertensive effects through modulation of SOD‐dependent antioxidant pathways. In experimental hypertension, tetrahydrocurcumin significantly reduced blood pressure while increasing SOD and GSH levels, attenuating renal fibrosis and reducing apoptosis. Lau et al. also reported reductions in proteinuria and improved renal architecture, supporting the compound's antioxidant and nephroprotective properties [67].

More recent studies have expanded this SOD‐centered framework. Pereira et al. reported that in L‐NAME–induced hypertension, Cur (50–100 mg/kg) significantly reduced SBP, prevented renal structural injury, restored serum creatinine to normal levels and increased renal SOD activity. Cur also inhibited matrix metalloproteinase (MMP)‐2 and MMP‐9 expression and activity, suppressed superoxide generation and reduced macrophage infiltration (ED‐1), thereby limiting oxidative stress–driven glomerular injury and inflammation [68]. Additionally, Cur nanoemulsion (self‐nanoemulsifying Cur, SNEC) demonstrated superior antihypertensive efficacy in deoxycorticosterone acetate (DOCA)‐salt hypertension models by inhibiting angiotensin‐converting enzyme (ACE) activity and reducing angiotensin II levels. SNEC restored SOD, GSH and CAT activities in cardiac tissue while reducing MDA and improving left ventricular contractility indices, including left ventricular end‐diastolic pressure (LVEDP), maximum rate of left ventricular pressure rise (Max dP/dt) and maximum rate of left ventricular pressure decline (Min dP/dt). These findings suggest that nanoCur enhances blood pressure control and oxidative stress reduction relative to free Cur, with SOD modulation acting as a central antioxidant mechanism within broader renin–angiotensin–aldosterone system (RAAS)‐related effects [69]. Despite consistent improvements in blood pressure and oxidative stress markers, including SOD activity, these findings are largely based on experimental hypertension models with limited clinical validation. Moreover, SOD is often evaluated alongside broader antioxidant changes, making it difficult to distinguish whether it acts as a primary regulator or as part of a coordinated redox response mediated by upstream pathways such as RAAS modulation or Nrf2 activation.

### 4.4 Cardiac Toxicity

Cardiac toxicity refers to structural and functional impairment of the myocardium arising from drugs, toxins or metabolic disturbances. Clinical features include chest pain, dyspnea, palpitations, peripheral edema and dizziness. Oxidative stress is a major mediator of chemotherapy‐induced cardiotoxicity, particularly in doxorubicin exposure, and reduced SOD activity contributes directly to ROS‐mediated cellular injury. In combined in vitro and in vivo studies, Cur (2.5 mg/kg, intraperitoneally) markedly mitigated doxorubicin‐induced cardiac injury by reducing MDA levels and increasing SOD and GPX expression. Cur also lowered cardiac biomarker expression, including atrial natriuretic peptide, B‐type natriuretic peptides and MHC‐β, underscoring its antioxidant and cardioprotective potential [70, 71]. Supporting these observations, Hamdy et al. showed that Cur profoundly mitigates gentamicin‐induced cardiac oxidative injury by normalizing cardiac SOD, CAT and GSH levels and reducing MDA accumulation. Cur reversed NF‐κB, IL‐1β, Keap1 and HMOX1 overexpression and restored the Bcl‐2/Bax ratio, thereby limiting apoptosis. Significant reductions in creatine kinase–myocardial band (CK‐MB), LDH and troponin I further confirmed Cur's cardioprotective effect, while histological evaluation showed reduced myofiber distortion, leukocyte infiltration and interstitial damage [63]. Together, these findings support the view that Cur limits cardiac toxicity primarily through restoration of SOD‐centered antioxidant balance, with anti‐inflammatory and anti‐apoptotic effects occurring as downstream consequences of improved redox control. While the protective effects of Cur against cardiac toxicity are well supported by preclinical data, these studies predominantly assess SOD as part of a generalized antioxidant response rather than through direct mechanistic manipulation. Consequently, although SOD restoration is consistently associated with reduced injury, its independent contribution to cardioprotection in toxic models requires further investigation.

### 4.5 Abdominal Aortic Aneurysm

Abdominal aortic aneurysm (AAA) is a life‐threatening vascular disorder and ranks among the leading causes of mortality in older adults, particularly men over 65. Although often silent, it may present with abdominal or back pain. AAA development is driven by chronic inflammation, proteolytic degradation of the extracellular matrix and oxidative stress [72, 73]. Cur has demonstrated significant AAA‐suppressive effects in experimental models through pathways closely linked to SOD restoration. Hao et al. showed that Cur (100 mg/kg) reduced aneurysm incidence and aortic diameter, suppressed macrophage infiltration and monocyte chemoattractant protein‐1 expression and markedly decreased ROS production. Cur also downregulated TNF‐α, MMP‐2 and MMP‐9 while increasing SOD levels. These effects were mediated through inhibition of the extracellular‐signal‐regulated kinase (ERK) 1/2 pathway [74]. Although few studies have specifically examined nanoparticle‐based Cur systems in AAA, evidence from related oxidative and inflammatory cardiovascular models suggests that Cur‐mediated enhancement of SOD activity, together with suppression of MMPs and inflammatory cytokines, may contribute broadly to stabilization of vascular structure and prevention of extracellular matrix degradation [63, 68]. Although available evidence suggests that Cur‐mediated SOD activation contributes to attenuation of oxidative stress and vascular remodelling in AAA, the number of studies remains limited. In addition, mechanistic insights are largely inferred from related cardiovascular models rather than directly validated in aneurysm‐specific systems, highlighting the need for more targeted investigations.

### 4.6 Diabetes Mellitus

**FIGURE 2:**
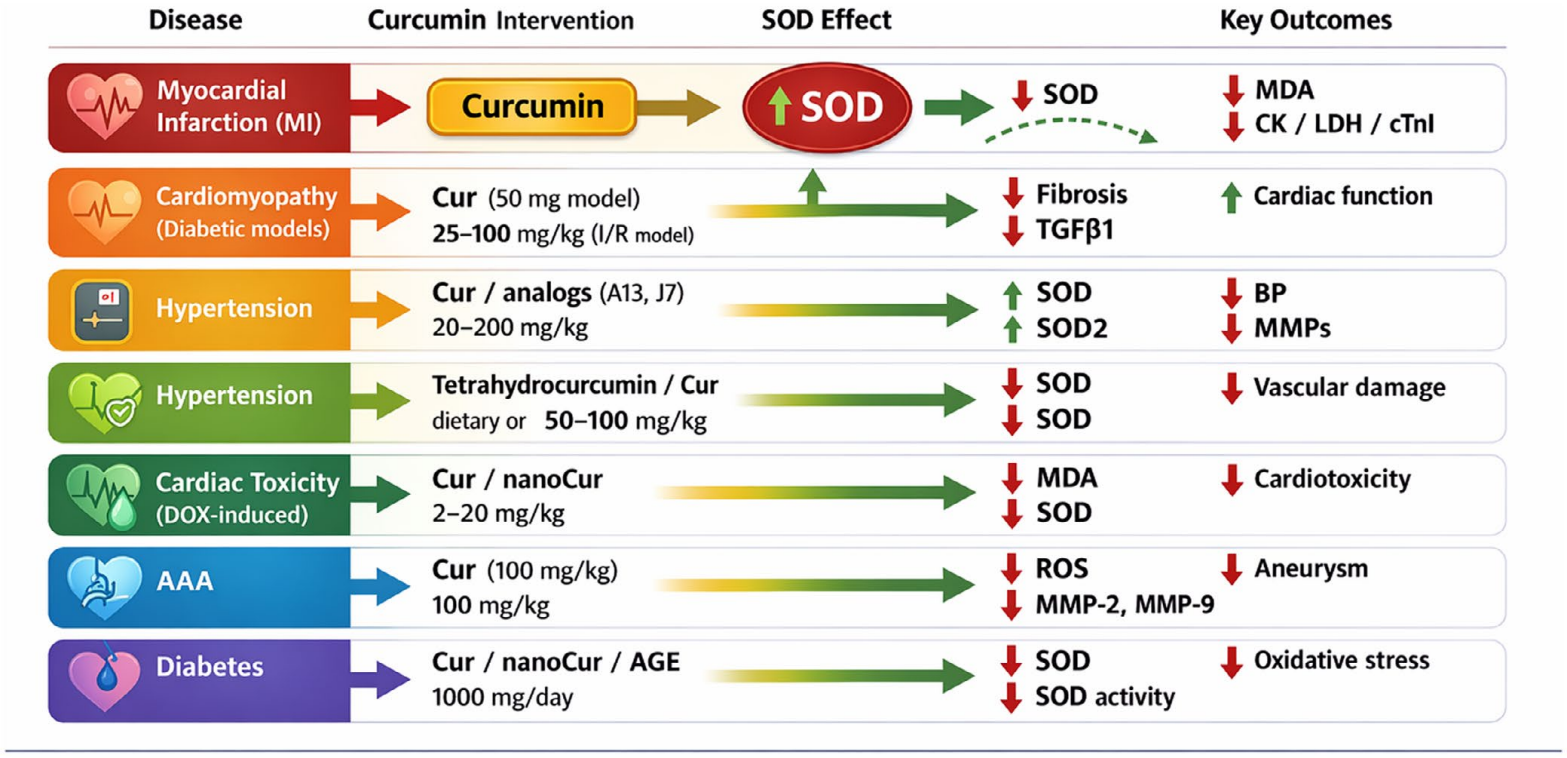
Disease‐oriented summary of curcumin‐mediated modulation of superoxide dismutase (SOD) and cardiovascular outcomes based on in vivo studies (Table 1). This schematic summarizes the disease‐specific effects of curcumin (Cur) on superoxide dismutase (SOD) across major cardiovascular disease (CVD) models derived from in vivo studies presented in Table 1. Curcumin administration consistently enhances SOD activity and/or expression, including cytosolic SOD1, mitochondrial SOD2 and extracellular SOD3, across diverse pathological conditions such as myocardial infarction (MI), cardiomyopathy, hypertension, cardiac toxicity, abdominal aortic aneurysm (AAA) and diabetes mellitus (DM). Upregulation of SOD is associated with attenuation of oxidative stress, as evidenced by reduced malondialdehyde (MDA) levels and decreased reactive oxygen species (ROS) accumulation, along with suppression of inflammatory signalling pathways and improved antioxidant defence systems. These redox improvements translate into reduced apoptosis, fibrosis and extracellular matrix remodelling, as well as improved mitochondrial function and vascular integrity. Consequently, curcumin‐mediated SOD activation contributes to favourable cardiovascular outcomes, including reduced infarct size, improved cardiac function, decreased blood pressure, attenuation of cardiotoxicity, inhibition of aneurysm progression and mitigation of diabetes‐associated oxidative complications. Arrows indicate direction of change (↑ increase; ↓ decrease). AAA, abdominal aortic aneurysm; BP, blood pressure; CK, creatine kinase; cTnI, cardiac troponin I; DM, diabetes mellitus; GPX, glutathione peroxidase; LDH, lactate dehydrogenase; MDA, malondialdehyde; MI, myocardial infarction; MMPs, matrix metalloproteinases; Nrf2, nuclear factor erythroid 2–related factor 2; ROS, reactive oxygen species; SOD, superoxide dismutase; SOD1, cytosolic superoxide dismutase; SOD2, mitochondrial superoxide dismutase; SOD3, extracellular superoxide dismutase.

Diabetes mellitus is characterized by insufficient insulin production or impaired insulin utilization. Classic symptoms include polyuria, polydipsia and polyphagia, although weight loss, impaired wound healing and increased susceptibility to infection are also common [75]. Inflammation and oxidative stress are major contributors to diabetic vascular injury and its cardiovascular complications. Hyperglycemia promotes ROS generation through glucose autoxidation, non‐enzymatic glycosylation of proteins and oxidative degradation of glycated proteins. The resulting oxidative burden, together with reduced antioxidant defences such as SOD, contributes to organelle damage, enzyme dysfunction and insulin resistance [76, 77]. Cur has been shown to restore redox balance in diabetes through modulation of SOD and downstream antioxidant pathways. In a clinical trial, Panahi et al. reported that curcuminoids (1000 mg/day) significantly increased serum SOD activity and total antioxidant capacity while lowering MDA levels, indicating meaningful reduction in oxidative stress burden in patients with type 2 diabetes mellitus [78]. Supporting this, preclinical evidence from renal and cardiac oxidative injury models shows that Cur restores SOD, GSH and CAT activity while reducing lipid peroxidation [63]. These observations are consistent with the concept that enhancement of SOD activity is a core mechanism through which Cur mitigates oxidative stress‐related diabetic cardiovascular complications, whereas broader anti‐inflammatory and metabolic benefits likely occur downstream of improved redox regulation (Table 1, Figure 2). Clinical and preclinical studies consistently demonstrate that Cur enhances SOD activity and ROS in diabetes; however, these findings are primarily based on systemic antioxidant measurements. Whether SOD modulation directly mediates cardiovascular protection in diabetic complications or reflects broader metabolic and inflammatory improvements remains to be clarified.

**TABLE 1:** The effect of Curcumin on CVD through SOD and related signalling pathways (in vivo *interventions*).

Animal Type	Disease	Intervention		Number of Animals		Treatment Duration	Results	Effect on SOD	References
		Case	Control	Case	Control				
Male Sprague–Dawley (SD) rat	STZ‐induced DM	DM DM + Cur (20 mg/kg, i.g.) DM + high‐dose A13 (20 mg/kg, i.g.) DM + low‐dose A13 (10 mg/kg, i.g.)	Nondiabetic	10–12 per group	8	10 weeks	Treatment with Cur analog A13: ↑ SOD & MDA; activated Nrf2/ARE; ↓ myocardial fibrosis	↑ SOD activity	[59]
Male ApoE−/− mice	Angiotensin II (Ang II)‐Abdominal Aortic Aneurysm (AAA)	AAA AAA + Cur (100 mg/kg/day,i.g.)	No treatment (Normal saline)	12 per group	12	4 weeks	↑ SOD; ↓ MMP‐2/9; ↓ ROS; ↓ MCP‐1; ERK1/2 inhibition; ↓ AAA incidence	↑ SOD activity	[74]
Female SD rat	Chronic kidney disease (CKD)	CKD CKD + 1% Tetrahydrocurcumin diet	No treatment	9–10 per group	6	9 weeks	Restored Cu/Zn‐SOD and GPX	↑ SOD activity	[67]
Male SD rat	Doxorubicin (DOX)‐induced cardiac toxicity	Cardiac toxicity+ Cur‐loaded magnetic hydrogel nanocomposite (5 mg/kg, i.p.)	Cardiac toxicity+ Cur (5 mg/kg), i.p.	5 per group	5	2 weeks	↑ SOD, ↑ GPX; ↓ MDA vs. DOX	↑ SOD activity	[70]
Male SD rat	STZ‐induced DM	DM DM + Cur (100 mg/kg/day, i.g)	No treatment	15 per group	10	4 weeks	↑ Akt phosphorylation; ↑ SOD; ↓ MDA	↑ SOD activity	[56]
Male C57BL/6 mice	STZ‐induced DM	DM DM+ Tetrahydrocurcumin (120 mg/kg/day, i.g.)	No treatment (Normal saline)	15 per group	15	12 weeks	↑ SOD2, ↑ GPX; ↓ MDA; ↓ α‐SMA, collagen I/III via TGFβ1/Smad3	↑ SOD2 activity	[57]
Male SD rat	DOX‐induced myocardial toxicity	Myocardial toxicity Myocardial toxicity + Cur (200 mg/kg,p.o.) Myocardial toxicity + Cur loaded mesoporous silica nanoparticles (200 mg/kg,p.o).	No treatment (Normal saline)	6 per group	6	2 weeks	↑ SOD, GSH, CAT; ↓ MDA; nanocarrier superior to Cur alone	↑ SOD activity	[58]
Male SD rat	STZ‐induced DM	DM DM + aged garlic extract (AGE) (500 mg/kg, p.o.) DM + nano Cur (300 mg/kg, p.o.)	Control (PBS)	18 per group	18	8 weeks	↑ Mn‐SOD gene expression in myocardium	↑ SOD activity	[60]
Male SD rat	STZ‐induced DM	DM DM + Cur (100 mg/kg/day, i,g,) DM + Cur (200 mg/kg/day, i,g,)	Vehicle	8–10 per group	8–10 per group	16 weeks	↑ SOD; ↓ MDA; ↓ NADPH oxidase components; ↓ TNF‐α, IL‐1β; ↑ Akt, ↑ GSK‐3β	↑ SOD activity	[61]
Male SD rat	DOX‐induced Cardiotoxicity	DOX Cur (200 mg/kg, p.o.) + DOX Cur (200 mg/kg, p.o.)	Normal saline (5 mL/kg)	6 per group	6	2 weeks	↑ SOD, ↑ CAT, ↑ GSH; ↓ MDA	↑ SOD activity	[62]
Male Wistar rat	MI	Cur (50 mg/kg,i.g.) Isoproterenol hydrochloride (ISO) (100 mg/kg, s.c.) ISO (100 mg/kg, s.c.) + Cur (50 mg/kg,i.g.)	Normal saline s.c.	6 per group	6	9 days	↑ SOD; ↓ MDA; ↓ CK, LDH, cTnI; ↓ apoptosis	↑ SOD activity	[47]
Male SD rat	MI (LAD ligation)	Cur‐PEG‐GQDs (5–10 mg/kg); Cur (3–15 mg/kg); PEG‐GQDs	MI alone	6 per group	6	2 weeks	↓ Infarct size; ↓ fibrosis; restored SBP, DBP, LVSP, ±dP/dt; ↓ MDA; ↑ GSH/GSSG	↑ SOD activity	[49]
Male SD rat	Ischaemia‐reperfusion injury	Cur (25, 50 and 100 mg/kg, i.p.) 30 min before operation	Normal saline	10 per group	20	1 day	↑ SOD, ↑ GSH; ↓ CK, LDH, MDA, NO; ↑ Bcl‐2; ↓ Bax	↑ SOD activity	[48]
Wistar rat	L‐NAME Hypertension	Cur (50 or 100 mg/kg/day, gavage)	L‐NAME alone	8 per group	8	2 weeks	↑ SOD; ↓ O_2_^−^; ↓ MMP‐2/9; ↓ ED1^+^ macrophages; ↓ creatinine; restored renal histology	↑ SOD activity	[68]
Nephrectomies rat	DOCA‐salt Hypertension	Nanocurcumin (60 or 90 mg/kg/day); Cur (60 or 90 mg/kg)	DOCA alone	7 per group	7	6 weeks	↓ SBP, DBP, MAP; ↓ LVEDP; ↑ contractility; ↓ ACE, ↓ Ang II;	↑ SOD activity	[69]

Abbreviations: AAA, abdominal aortic aneurysm; ACE, angiotensin‐converting enzyme; AGE, aged garlic extract; Ang II, angiotensin II; ARE, antioxidant response element; CAT, catalase; CK, creatine kinase; CKD, chronic kidney disease; cTnI, cardiac troponin I; Cur, curcumin; DBP, diastolic blood pressure; DM, diabetes mellitus; DOX, doxorubicin; ERK, extracellular signal‐regulated kinase; GPX, glutathione peroxidase; GSH, glutathione; GSH/GSSG, reduced‐to‐oxidized glutathione ratio; HO‐1, heme oxygenase‐1; i.g., intragastric; i.p., intraperitoneal; ISO, isoproterenol; LAD, left anterior descending artery; LDH, lactate dehydrogenase; LVEDP, left ventricular end‐diastolic pressure; LVSP, left ventricular systolic pressure; MAP, mean arterial pressure; MDA, malondialdehyde; MI, myocardial infarction; Nrf2, nuclear factor erythroid 2–related factor 2; p.o., oral; SBP, systolic blood pressure; SOD, superoxide dismutase; STZ, streptozotocin.

## 5 Critical Evaluation of SOD‐Centered Evidence Across CVD Models

Although a substantial body of evidence demonstrates that Cur enhances SOD activity across CVD models, the overall strength of this evidence remains predominantly preclinical and methodologically heterogeneous. Most studies included in this review are based on rodent models or immortalized cell lines, often with limited sample sizes and relatively short intervention durations, which may restrict translational applicability to human disease. Furthermore, experimental variability in disease induction models, Cur formulations (free vs. nanodelivery), dosing strategies and outcome measures complicates direct comparison across studies. A critical limitation is that SOD is frequently assessed as a global antioxidant endpoint, typically measured as total enzymatic activity or protein expression, rather than through isoform‐specific or mechanistic interrogation. Only a limited number of studies distinguish between cytosolic SOD1, mitochondrial SOD2 and extracellular SOD3, despite their distinct biological roles in regulating compartmentalized redox signalling. Moreover, few investigations employ genetic manipulation, selective inhibition or gain‐of‐function approaches to directly establish whether SOD modulation is causally responsible for the observed cardioprotective effects. Consequently, while increased SOD activity is consistently associated with reductions in ROS, lipid peroxidation (e.g., malondialdehyde, MDA) and downstream cellular injury, it remains uncertain whether SOD functions as a primary mechanistic driver or as a surrogate marker of broader redox restoration. Indeed, many of the reported effects of Cur are mediated through upstream regulatory pathways, including Nrf2, NF‐κB, PI3K/AKT and SIRT1, which collectively influence antioxidant defence systems, including SOD, GSH, GPX and CAT. Additionally, the lack of standardized endpoints—particularly with respect to SOD isoform activity, tissue‐specific measurements and functional cardiovascular outcomes, further limits the ability to define SOD as a central mechanistic node in Cur‐mediated cardioprotection. Importantly, no studies to date have systematically evaluated SOD modulation as a primary therapeutic target in clinically relevant models or human populations. Taken together, these findings suggest that while SOD modulation represents a consistent and biologically plausible component of Cur's antioxidant effects, current evidence primarily supports an associative rather than causative role. Addressing these limitations through isoform‐specific analyses, standardized experimental designs and clinically relevant models will be essential to establish the mechanistic and therapeutic significance of SOD in CVD. These considerations directly inform the interpretation of Cur's translational potential and highlight the need for cautious extrapolation of preclinical findings to human disease contexts.

## 6 Conclusion & Future Perspectives

CVD arise from complex and interrelated pathological processes, including chronic inflammation, oxidative stress, endothelial dysfunction and progressive cellular degeneration. Among these, disruption of redox homeostasis plays a central role in driving disease initiation and progression. SOD, as a key enzymatic defence against ROS, represents a critical regulator of oxidative balance, and its impairment has been implicated across a broad spectrum of cardiovascular conditions. The evidence synthesized in this review indicates that Cur exerts protective effects in multiple experimental models of CVD, including myocardial infarction, cardiomyopathy, hypertension, cardiac toxicity, abdominal aortic aneurysm and diabetes mellitus. In both in vitro and in vivo settings, Cur consistently enhances antioxidant capacity, as reflected by increased activity of SOD, GSH, GPX and CAT, alongside reductions in oxidative stress markers such as MDA. These biochemical effects are accompanied by modulation of key signalling pathways, including Nrf2, NF‐κB and PI3K/AKT, resulting in attenuation of inflammation, apoptosis, fibrosis and mitochondrial dysfunction. Despite these promising preclinical findings, translation into clinical application remains limited. As discussed in the clinical evidence section, studies directly evaluating Cur in patients with established cardiovascular disease as the primary diagnosis are scarce. Most available human data originate from cardiometabolic or non‐cardiovascular conditions, where Cur has been shown to improve systemic oxidative stress markers, including SOD activity, without directly assessing cardiovascular endpoints or mechanistic pathways. Importantly, no clinical studies have yet demonstrated that SOD modulation represents a causal mechanism underlying cardiovascular benefit in humans. An additional consideration in the translational interpretation of these findings is the potential divergence between rodent and human redox biology. Differences in SOD isoform expression, tissue distribution and regulation of antioxidant pathways may influence the extent to which preclinical results can be extrapolated to human cardiovascular disease. Moreover, variations in metabolic rate, immune response and oxidative stress dynamics between species may alter curcumin's pharmacological effects. As a result, improvements in SOD activity observed in experimental models may not directly translate to equivalent therapeutic outcomes in humans. These interspecies differences represent a critical knowledge gap and underscore the need for mechanistically informed clinical studies.

Another major challenge lies in Cur's low intrinsic bioavailability, driven by poor aqueous solubility, rapid metabolism and limited systemic distribution. Although emerging delivery systems, such as nanoemulsions, liposomal formulations and graphene‐based nanocarriers, have demonstrated improved pharmacokinetic profiles and enhanced biological activity, further optimization and standardization are required before these approaches can be translated into routine clinical use. Future research should focus on several key priorities. First, mechanistic studies are needed to delineate the specific roles of SOD isoforms (SOD1, SOD2 and SOD3) in Cur‐mediated cardiovascular protection, particularly in the context of ferroptosis, autophagy, apoptosis and mitochondrial energetics. Second, well‐designed randomized controlled trials in defined CVD populations are essential to evaluate clinical efficacy, establish optimal dosing strategies and assess long‐term safety. Third, the development of predictive biomarkers, such as oxidative stress signatures or SOD‐related activity profiles, may enable patient stratification and improve therapeutic targeting. Finally, investigation of potential synergistic effects between Cur and established cardiovascular therapies may further enhance translational relevance. In summary, current evidence supports Cur as a biologically active compound capable of modulating redox homeostasis through SOD‐associated pathways in preclinical models of cardiovascular disease. However, substantial mechanistic clarification and rigorous clinical validation are required to determine whether these effects can be translated into effective therapeutic strategies in human cardiovascular medicine.

## Author Contributions

**Danial Khayatan:** writing – original draft. **Seyed Mehrad Razavi:** writing – original draft. **Zahra Najafi Arab:** writing – original draft. **Amirhossein Niknejad:** writing – original draft. **Yasamin Hosseini:** writing – original draft. **Ayeh Sabbagh Kashani:** writing – original draft. **Saeideh Momtaz:** writing – original draft. **Tannaz Jamialahmadi:** writing – original draft. **Prashant Kesharwani:** conceptualization, writing – review and editing, supervision. **Amir Hossein Abdolghaffari:** writing – review and editing. **Amirhossein Sahebkar:** conceptualization, writing – review and editing, supervision.

## Funding

The authors have nothing to report.

## Conflicts of Interest

The authors declare no conflicts of interest.

## Data Availability

Data sharing not applicable to this article as no datasets were generated or analysed during the current study.
